# Dyskinesia-hyperpyrexia syndrome in Parkinson’s disease: a systematic review

**DOI:** 10.1007/s10286-021-00801-w

**Published:** 2021-04-07

**Authors:** Miao Wang, Wei Wang, Zhongbao Gao, Xi Yin, Tong Chen, Ziying Jiang, Zhenfu Wang

**Affiliations:** Geriatric Neurological Department of the Second Medical Center, National Clinical Research Center for Geriatric Diseases, Chinese People’s Liberation Army General Hospital, Beijing, China

**Keywords:** Dyskinesia-hyperpyrexia syndrome, Parkinson hyperpyrexia syndrome, Serotonin syndrome, Clinical practice

## Abstract

**Purpose:**

Dyskinesia-hyperpyrexia syndrome (DHS) is a rare but life-threatening disease. The clinical manifestations of this syndrome overlap substantially with Parkinson hyperpyrexia syndrome and serotonin syndrome and are often confused by clinicians. The purpose of this review was to enable clinicians to recognize this syndrome and thereby reach a correct diagnosis and provide optimal treatments to improve prognosis in clinical practice.

**Methods:**

Using the methodology described in the Preferred Reporting Items for Systematic Reviews and Meta-Analyses (PRISMA) statement, we conducted a literature search of the PubMed, Embase, and MEDLINE databases using keywords in titles and abstracts of published literature. Quality assessment was performed using the modified Newcastle-Ottawa scale.

**Results:**

A total of 11 patients obtained from nine publications were included in this systematic review. All of the cases occurred in patients with advanced Parkinson's disease (PD) of long disease duration. High ambient temperature was the most common trigger of this syndrome. Hyperpyrexia and dyskinesias were present in all cases. The consciousness disturbances of this syndrome included confusion, hallucination, and lethargy or stupor. Autonomic dysfunction (except for hyperpyrexia) is uncommon in DHS, and only two patients presented with tachycardia. The treatment of this syndrome included supportive interventions (including rehydration, anti-pyretic and anti-infection treatments, and maintaining electrolyte balance), dopaminergic drug reduction and sedation. Two patients died due to DHS.

**Conclusions:**

We summarized the triggers, clinical features, and treatments of all reported dyskinesia-hyperpyrexia syndrome cases, proposed guiding diagnostic criteria, and established a flow chart to guide diagnoses to quickly identify these three syndromes in clinical practice.

**Supplementary Information:**

The online version contains supplementary material available at 10.1007/s10286-021-00801-w.

## Introduction

Parkinson’s disease (PD) is a chronic, progressive movement disorder. However, PD patients sometimes develop acute complications that are serious or even life-threatening, requiring prompt medical attention. Hyperpyrexia is a common cause of emergency admission in PD patients [1]. When a PD patient is admitted to the emergency room (ER) because of acute hyperpyrexia, infection is first considered. Parkinson hyperpyrexia syndrome (PHS, also known as neuroleptic malignant-like syndrome) and serotonin syndrome (SS) [2, 3] also need to be considered. In the past 10 years, dyskinesia-hyperpyrexia syndrome (DHS), a rare but potentially fatal complication in PD, has been reported in PD patients [4]. The clinical manifestations of DHS, PHS, and SS overlap substantially and are often confused by clinicians. However, the triggers and treatments of these syndromes remain distinct. If the right diagnosis is not made, timely and optimal treatment cannot be delivered. This may lead to disastrous consequences for patients with these syndromes. We performed a systematic review to summarize triggers, clinical features, treatments, and outcomes of DHS. Next, we proposed guiding diagnostic criteria for DHS for the first time. Finally, we elucidated the similarities and differences between PHS, DHS, and SS, establishing a flow chart to guide diagnosis, to quickly identify these three syndromes in clinical practice.

## Materials and methods

The Preferred Reporting Items for Systematic Reviews and Meta-Analyses (PRISMA) guidelines were followed during this systematic review in order to ensure the transparency and completeness of the review process.

### Search strategies

A literature search of the PubMed, Embase, and MEDLINE databases was conducted on 17 January 2021 on the subject of DHS, with three search terms. Term A was “Parkinson’s disease” OR “Parkinsonism” OR “Parkinson disease.” Term B was “dyskinesia” OR “dyskinesias” OR “hyperkinetic.” Term C was “fever” OR “hyperpyrexia” OR “pyrexia” OR “pyrexias.” An additional search of the PubMed and MEDLINE databases was conducted using the keywords “dyskinesia-hyperpyrexia syndrome.”

### Inclusion and exclusion criteria

Articles were included if they met the following criteria: (1) written in English, and (2) reported on PD patients with acute dyskinesia and hyperpyrexia. Articles were excluded if they met the following criteria: (1) duplicate articles, or (2) full text could not be obtained. Two authors (Wang Miao, Wang Wei) independently screened all titles and abstracts, as well as the full texts, for manuscript selection. Those found to meet either of the exclusion criteria were removed, and any conflicts were settled by consensus during an in-person meeting in which the abstracts were reread.

### Risk of bias (methodological quality) assessment of individual studies

The quality of the included studies was evaluated using the modified Newcastle–Ottawa scale [5, 6]. This tool consists of five items, presented as questions: (a) Did the patients constitute all of the cases at the medical center? (b) Was the diagnosis correctly made? (c) Were other important diagnoses excluded? (d) Were all important data cited in the report? (e) Was the outcome correctly ascertained? For each question, a binary response indicated whether or not the item was suggestive of bias. We considered the quality of the report as good (low risk of bias) when all five criteria were fulfilled, moderate when four were fulfilled, and poor (high risk of bias) when three or fewer were fulfilled. The same two authors (Wang Miao, Wang Wei) assessed the risk of bias of the included studies, with a discussion in the case of disagreement.

### Data extraction

Two authors (Wang Miao, Wang Wei) then, independently, extracted data from all relevant reports [4, 7–13] using a standardized form. The following data were extracted from each manuscript: author, year of publication, age and gender of the patient, the duration of PD, the season of DHS onset, the possible triggers, the anti-Parkinsonism medications taken before DHS, the symptoms and complications of DHS, the duration of DHS, the treatment of DHS and the outcome of DHS. The collected data were entered into a Microsoft Excel spreadsheet (version 11.0; Microsoft Corporation, Redmond, WA).

## Results

**Fig. 1:**
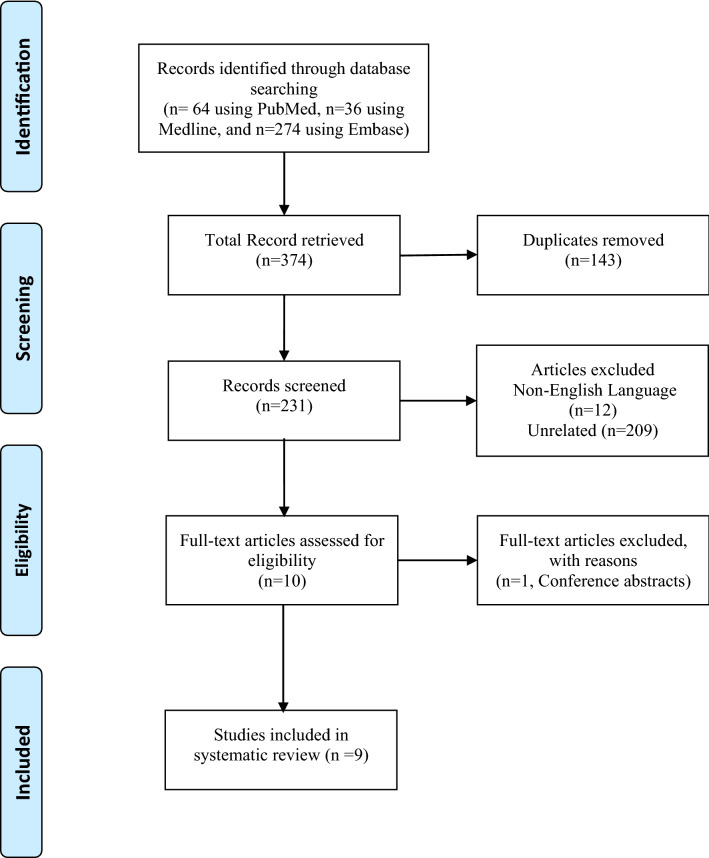
PRISMA flow diagram

Figure 1 represents a flow chart of the study selection. All abstracts and titles from 374 publications retrieved from the electronic database search were thoroughly screened. Among these, 231 publications were selected for title and abstract review. A total of 11 patients obtained from nine publications met the inclusion criteria for analysis. All nine publications were case reports. The publication dates of the included studies ranged from 2010 to 2021. The results of the risk of bias assessment of the included studies are shown in Table 1. Six publications were identified as having low risk of bias (66.7%), and three moderate risk (33.3%). For item (a), it was not mentioned in the publications whether the reported patient(s) represented all cases at the medical center, and we assumed that the authors had reported all known cases in their center, indicating the rarity of DHS.

**Table 1:**
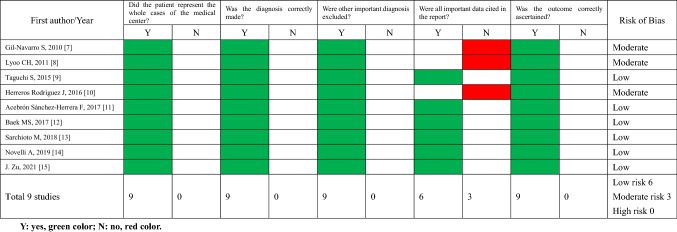
Risk of bias assessment of included studies

*Y* yes, green color; *N* no, red color

The clinical features of all DHS cases are displayed in Table 2. We identified a total of 11 patients who experienced 19 episodes of DHS (one patient experienced eight episodes [9], one experienced two episodes [11], and the remaining eight experienced one episode), with a female predominance (eight female, three male) and a mean age of 72.5 years (range 62–80 years). The mean duration of PD was 18.8 years (range 10–34 years). Among the 19 episodes of DHS, 13 occurred in the summer, two occurred in the spring, and one occurred in the autumn (the onset season of three DHS episodes was not mentioned [4, 7]). The triggers of DHS were high ambient temperature (13 episodes) [9, 10, 12, 13], infection (six episodes) [11–13], trauma (two episodes) [11] and dopaminergic drug dosage increase (three episodes) [7, 10, 14] or a change in dopaminergic drug form (one episode) [8] (the trigger of one DHS episode was not mentioned [4]). The mean levodopa equivalent daily dose (LEDD) before a DHS attack was 1315.6 mg (range 675–2528 mg) and the LEDD before two DHS episodes were not mentioned [9]. All DHS episodes were characterized by hyperpyrexia (mean body temperature peak, 39.9 °C; range 38.2–42 °C) and generalized dyskinesias. A total of 9 out of 19 DHS episodes manifested consciousness disturbances including confusion, hallucination and lethargy or stupor [4, 8, 10–14]. The serum creatine kinase (CK) levels were elevated in 16 episodes of DHS (mean CK peak, 6973.2 IU/L; range 211–35,000 IU/L), serum CK levels were normal in one episode [9] and serum CK levels were not mentioned in two episodes [9]. The complications of DHS included pneumonia [11, 12], electrolyte disturbance [12, 13], acute renal injury [7, 11, 12] and respiratory failure [12]. DHS treatment included supportive interventions (including rehydration, anti-pyretic and anti-infection treatments, and maintaining electrolyte balance), dopaminergic drug reduction and sedation. Two patients died due to DHS [12].

**Table 2:** The clinical features of all DHS cases

Report	A/G/YOD	Season	Possible triggers	Anti-Parkinsonism medications before DHS	Levodopa equivalent daily dose	Symptoms and complications	Duration of DHS	Treatment	Outcome
Gil-Navarro, 2010 [4]	68/F/12	NA	NA	Levodopa/carbidopa/entacapone (5 daily doses of 150/50/200 mg) Pramipexole 4 mg per day Amantadine 200 mg per day	1680 mg	Peak body temperature 41.2 °C Generalized dyskinesias Confusion and hallucination CK = 1455 IU/L Tachycardia	Within 6 days	Intravenous fluids Antipyretic Pramipexole tapered off Quetiapine 25 mg	Recovery
Lyoo, 2011 [7]	74/M/17	NA	Dopaminergic drug dose increase (levodopa 1050 mg increased to 3400 mg)	12 tablets of levodopa/carbidopa 250/25 mg per day 4 tablets of levodopa/benserazide 100/25 mg per day	3400 mg	Peak body temperature 38.2 °C Generalized dyskinesias Consciousness was normal CK = 24,651 IU/L Acute renal injury (176.8 µmol/L)	5 days	Sedative Dopaminergic drug was stopped	Recovery
Taguchi, 2015 [8]	70/F/13	Autumn	Dopaminergic drug form change (pramipexole IR to ER)	Levodopa 600 mg per day Pramipexole (ER) 3 mg per day Selegiline 5 mg per day	950 mg	Peak body temperature 40.3 °C Generalized dyskinesias Confusion and hallucination CK = 35,000 IU/L Tachycardia	7 days	Intravenous fluids Antipyretic Reduced dopaminergic drugs	Recovery
Herreros, 2016 [9]	74/F/16	Summer (8 episodes from 2007 to 2009)	High ambient temperature	NA	1390 mg	Peak body temperature 39 °C Generalized dyskinesias Consciousness was normal CK = 845 IU/L	NA	After the summer of 2009, the patient started to receive an LCIG (1310 mg/d)	Recovery
			High ambient temperature	NA	975 mg	Peak body temperature 40 °C Generalized dyskinesias Consciousness was normal CK = 1008 IU/L	NA		Recovery
			High ambient temperature	NA	975 mg	Peak body temperature 39.6 °C Generalized dyskinesias Consciousness was normal CK = 2509 IU/L	NA		Recovery
			High ambient temperature	NA	826 mg	Peak body temperature 38.8 °C Generalized dyskinesias Consciousness was normal CK = 211 IU/L	NA		Recovery
			High ambient temperature	NA	826 mg	Peak body temperature 38.3 °C Generalized dyskinesias Consciousness was normal CK = 178 IU/L	NA		Recovery
			High ambient temperature	NA	670 mg	Peak body temperature 40.2 °C Generalized dyskinesias Consciousness was normal CK = 257 IU/L	NA		Recovery
			High ambient temperature	NA	NA	Peak body temperature NA Generalized dyskinesias Consciousness was normal CK = NA	NA		Recovery
			High ambient temperature	NA	NA	Peak body temperature NA Generalized dyskinesias Consciousness was normal CK = NA	NA		Recovery
Acebrón Sánchez-Herrera, 2017 [10]	66/F/16	Summer	High ambient temperature/dopaminergic drug dose increase	LCIG (1450 mg/d and 362.5 mg/day) Amantadine 200 mg per day Ropinirole (ER) 8 mg per day Safinamide 100 mg per day	1810 mg	Peak body temperature 40.2 °C Generalized dyskinesias Confusion and hallucination CK = 7177 IU/L	4 days	Intravenous fluids Antipyretic Sedative LCIG reduced Amantadine, ropinirole and safinamide were stopped	Recovery
Baek, 2017 [11]	74/F/23	Spring (72 years old)	Trauma/infection	Levodopa 375 mg per day Pramipexole (ER) 1 mg per day Amantadine 200 mg per day	675 mg	Peak body temperature 40.3 °C Generalized dyskinesias Confusion and hallucination CK = 10,230 IU/L Acute renal injury (142 µmol/L) Aspiration pneumonia	4 days	Sedative Antibiotic LCIG reduced Amantadine and pramipexole were stopped	Recovery
		Spring (74 years old)	Trauma/infection	Levodopa 500 mg per day Pramipexole (ER) 1 mg per day Amantadine 300 mg per day	900 mg	Peak body temperature 39.2 °C Generalized dyskinesias Confusion CK = 6670 IU/L Acute renal injury (108 µmol/L)	1 day	Intravenous fluids Antipyretic Levodopa reduced to 300 mg and pramipexole stopped	Recovery
Sarchioto, 2018 [12]	80/M/20	Summer	High ambient temperature/infection	LCIG 1500 mg per day Pramipexole 1 mg per day Amantadine 200 mg per day Sertraline 50 mg per day	1550 mg	Peak body temperature 42 °C Generalized dyskinesias Confusion and lethargy CK = 16,040 IU/L Acute renal failure (186.5 µmol/L) Pneumonia	5 days	Intravenous fluids Antipyretic Antibiotics Pramipexole and amantadine stopped LCIG reduced to 700 mg	Death
	76/F/10	Summer	High ambient temperature/infection	LCIG 1200 mg per day Pramipexole 1 mg per day	1060 mg	Peak body temperature 41 °C Generalized dyskinesias Stupor CK = 2967 IU/L Pneumonia Respiratory failure	1 day	Antibiotics	Death
	79/F/30	Summer	High ambient temperature/infection	LCIG 1250 mg per day	1000 mg	Peak body temperature 39.5 °C Generalized dyskinesias CK = 1967 IU/L Pneumonia Acute renal injury (175 µmol/L)	Within 10 days	Intravenous fluids Antibiotics LCIG reduced to 675 mg	Recovery
Novelli, 2019 [13]	62/M/34	Summer	High ambient temperature/infection	STN-DBS (R-3 V, L-2.6 V) Levodopa/carbidopa 250/25 mg 8 daily doses per day Entacapone 200 mg 8 daily doses per day	2528 mg	Peak body temperature 40.7 °C Generalized dyskinesias Confusion CK = 4891 IU/L Tachycardia	2 days	Intravenous fluids Antipyretic agents Antibiotics DBS reduced to 1 V bilaterally Levodopa/carbidopa 125/12.5 mg 6 daily doses per day Entacapone 200 mg 6 doses per day	Recovery
Zu, 2021 [14]	76/F/16	NA	Dopaminergic drug dose increase (levodopa/carbidopa sustained-release tablets from 600/150 mg increased to 750/75 mg)	Levodopa/carbidopa sustained-release tablets 750/75 mg per day Entacapone 300 mg per day Rasagiline 1 mg per day	1150 mg	Peak body temperature 40.2 °C Generalized dyskinesias Unconsciousness CK = 2489 IU/L Tachycardia	Within 10 days	Reduced dopaminergic drugs	Recovery

*A* age, *G* gender, *YOD* years of Parkinson’s disease, *M* male, *F* female, *NA* not available, *IR* immediate-release, *ER* extended-release, *LCIG* levodopa-carbidopa intestinal gel, *STN-DBS* subthalamic nucleus deep brain stimulation, *CK* creatine kinase,

## Discussion

About 30–40% of PD patients who have been treated with levodopa for more than 5 years are observed to develop levodopa-induced dyskinesia of varying degrees of severity [15, 16]. Generally, these cases of dyskinesia are benign and can be treated in an outpatient setting. However, these dyskinesias may sometimes become severe and life-threatening, as they can lead to rhabdomyolysis, acute renal failure and respiratory distress. Dyskinesia associated with hyperpyrexia was first described in a 68-year-old advanced PD patient by Gil-Navarro and Grandas in 2010 [7]. They first proposed the term DHS. To date, a total of 11 cases of DHS have been reported in 9 publications (Table 2). In our review, all DHS cases occurred in patients with advanced PD of long disease duration, motor symptom fluctuation, and a high daily dose of dopaminergic medication. Furthermore, we observed that most patients were female (seven female and three male). These gender differences might be caused by female hormonal patterns increasing the individual dyskinetic sensitivity to levodopa [17] and by the lighter weight of most females, who have higher levodopa plasma levels after drug administration than their heavier male counterparts [18].

**Fig. 2:**
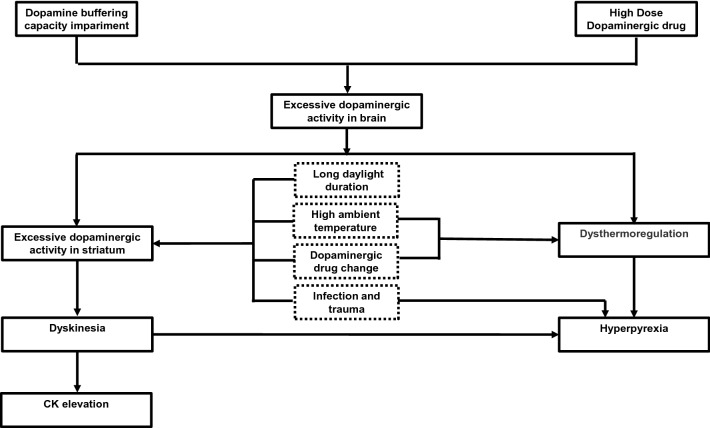
The possible pathophysiological mechanism underlying DHS

The pathophysiological mechanisms underlying DHS are not properly understood, and we speculated a possible pathophysiological mechanism of DHS in Fig. 2. We observed that summer or high ambient temperature was the most common trigger of DHS (13 of 19 DHS episodes). It was also reported that advanced PD patients developed hyperpyrexia without dyskinesia and akinesia in high ambient temperatures [19]. The dopaminergic system of the nigrostriatal system and hypothalamus plays an important role in thermoregulation. Fundamental research in mice has shown that when the ambient temperature is < 22  °C, stimulating the dopaminergic system in the nigrostriatal system and hypothalamus can lead to body temperature decreasing (by reducing metabolism and dilating skin blood vessels). However, when the ambient temperature is > 30  °C, stimulating the dopaminergic system in the nigrostriatal system and hypothalamus can lead to body temperature increasing (by increasing metabolism and constricting skin blood vessels) [20, 21]. In addition, further research has shown that injecting dopaminergic receptor antagonists at high ambient temperatures (43 °C) could delay the body temperature rise in mice [22]. The above research suggests that excessive dopaminergic activity might lead to body temperature increases at high ambient temperature. In advanced PD patients, dopamine buffering capacity impairment is widely observed due to nigrostriatal dopaminergic neuronal degeneration (impairing the compensatory synthesis, release, storage and reuptake of dopamine) [22] and postsynaptic dopamine receptor dysfunction [23]. After administration of high-dose dopaminergic drugs, the dopaminergic levels in the brain were dramatically increased in advanced PD patients due to impaired dopamine buffering capacity. Thus, we speculated that, in a high ambient temperature in summer, it is easier to trigger hyperpyrexia in advanced PD patients with high-dose dopaminergic drugs. In addition, hyperpyrexia was also related to an increase in thermogenesis caused by severe dyskinesia. At present, there is no unequivocal evidence that summer/high ambient temperatures are associated with dyskinesia. However, in clinical practice, we have also observed that the severity of symptoms in PD patients may change with the change in seasons. Symptoms appear to be less pronounced when the climate is warmer in the summer months and worse in winter (when there is also less daylight). Furthermore, in mice models, the functioning of the substantia-nigra pars compacta has been demonstrated to be heat sensitive [25]. Cooling caused low activity of the substantia nigra pars compacta, while warming induced the opposite effects. This suggests that a high ambient temperature in summer might increase presynaptic dopaminergic activity. Moreover, further research demonstrated that daylight duration is positively correlated with the activity of the D2/D3 receptors in the striatum of the human brain, and that daylight duration in summer is significantly greater than that in other seasons [26]. This suggests that long periods of daylight in the summer might increase postsynaptic dopaminergic activity. We speculated that in summer, under high ambient temperature (presynaptic dopaminergic activity increasing) and long daylight duration (postsynaptic dopaminergic activity increasing), it is easier to trigger dyskinesia in advanced PD patients with high-dose dopaminergic drugs. Infection and trauma are important factors of DHS and may induce DHS independent of summer and drug change [11]. The cytokines released during an infection and trauma might affect neural transmission and might lead to striatal dopaminergic hyperactivity [11, 27], and then induce dyskinesia under high-dose dopaminergic drugs.

The clinical features of DHS consist of hyperpyrexia (a manifestation of autonomic dysfunction, but since hyperpyrexia is a major symptom of DHS, we list it separately), severe and generalized dyskinesia, consciousness disturbance, autonomic dysfunction, and CK elevation. Dyskinesia and hyperpyrexia are seen in all DHS patients. Dyskinesia is continuous, severe, and generalized, and usually occurs prior to hyperpyrexia. Therefore, in the early stages of DHS, some patients may only present with dyskinesia without hyperpyrexia. The increased CK level is believed to be secondary to severe dyskinesia, ranging from hundreds to 35,000 IU /L. However, not all DHS patients manifest with CK elevation. We noticed that in a patient with eight episodes, reported by Rodriguez [9], the CK level increased in seven episodes, while the CK level was normal in one episode. Therefore, we suggested that CK elevation was not essential for DHS. Consciousness disturbance occurred in 9 out of 19 episodes, usually in a mild form, and manifested as confusion and hallucinations, which might be caused by dopaminergic hyperactivity in the mesocorticolimbic system. Only three patients manifested with consciousness level reduction (stupor or lethargy, unconsciousness) [12, 14]. Autonomic dysfunction (except for hyperpyrexia) is uncommon in DHS, and only two patients manifested with tachycardia. Currently, there are no unified diagnostic criteria for DHS. According to the symptoms summarized in all DHS cases, we proposed guiding diagnostic criteria for DHS, as shown in Table 3. Furthermore, it should be emphasized that the sensitivity and specificity of this diagnostic criteria need to be further confirmed by more cases in the future. Promptly reducing the dopaminergic medication was the most effective treatment, and dyskinesia could be improved in a short period of time (within a few days to 2 weeks). Novelli reported a patient with STN-DBS who developed DHS, in addition to reducing the dopaminergic medication; they also reduced DBS, and the patient recovered within 2 days [13]. In patients with refractory dyskinesias, sedation has been shown to be effective [7, 12, 13]. Supportive care, in the form of intravenous fluid replacement, anti-pyretic drugs, antibiotics, and maintenance of electrolyte balance, is also important in the treatment of DHS.

**Table 3:** Criteria for Guidance in the Diagnosis of DHS

Essential feature	Core feature	Supportive features
Severe and generalized dyskinesia	Hyperpyrexia	CK elevation or rhabdomyolysis
		Consciousness disturbance
		Autonomic dysfunction

The patient with acute-onset presented essential features and core features, with or without supportive features, and the diagnosis could be DHS. The patient presented essential feature with 2 supportive features, and the possible diagnosis could be DHS.

**Fig. 3:**
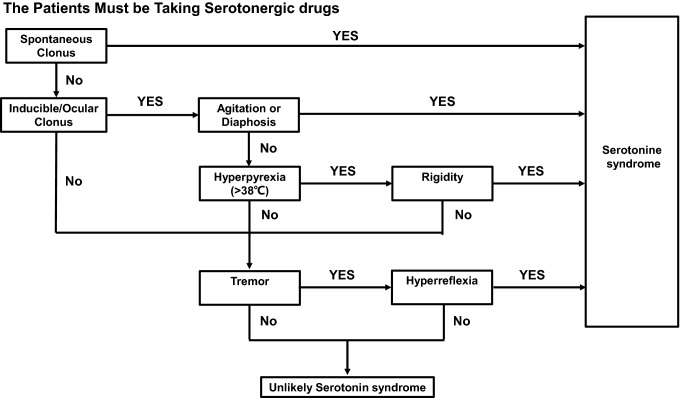
The Hunter serotonin toxicity criteria

Although when PD patients develop acute hyperthermia, the cause most frequently will be systemic infection, the possibility of Parkinson hyperpyrexia syndrome (PHS), DHS, and serotonin syndrome should be routinely considered and additional features suggestive of these disorders sought, since early diagnosis of these potentially lethal disorders is vital. PHS was first reported in 1981 by Toru et al. [28]. They reported a PD patient who presented with hyperpyrexia, autonomic instability, muscular rigidity, conscious dysfunction, and elevated serum CK, clinically similar to neuroleptic malignant syndrome). However, this patient had not been exposed to neuroleptics, but he abruptly ceased taking high-doses of anti-parkinsonian medications prior to the onset of symptoms. Except for obvious dosage reductions, PHS may also be induced by infection, high ambient temperature and STN-DBS device dysfunction [29–31]. The clinical manifestations of PHS include hyperpyrexia, worsening parkinsonism with severe rigidity, consciousness disturbance, autonomic dysfunction, and CK elevation [29]. Reinstituting levodopa or dopamine agonists and restarting STN-DBS are the most effective treatments [32]. SS is a drug-induced syndrome caused by the over-stimulation of postsynaptic serotonin receptors [33]. Selective serotonin reuptake inhibitors (SSRI) are the most frequent cause of SS [34]. Depression is a common nonmotor symptom of PD. Combination of monoamine oxidase B (MAO-B) inhibitors (selegiline and rasagiline) and SSRI increases the risk of SS in PD patients. SS usually occurs minutes or hours after the initial use of medication or dosage change, usually within 6 h [31]. The clinical presentation of SS includes rigidity, akinesia, hyperreflexia, autonomic nervous system excitation, and altered mental state [34]. In 2003, Dunkley et al. developed a new diagnostic criterion known as Hunter serotonin toxicity criterion (Fig. 3), with high specificity (97%), high sensitivity (84%) and high accuracy (95.6%) [35]. Discontinuation of all serotonergic drugs was the most effective treatment and SS usually resolved within 24 h.

**Fig. 4:**
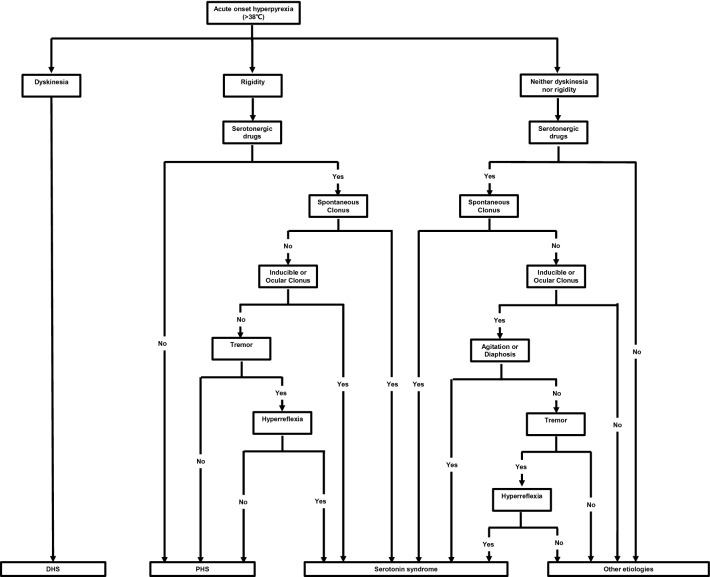
The diagnostic flow chart of DHS, PHS and SS

According to the above, the three syndromes share similar clinical presentations, such as hyperpyrexia, neuromuscular signs, autonomic dysfunctions, and consciousness disturbances, thereby remaining difficult to distinguish. However, there are some clinical clues to help with their differentiation. We summarize the differences in the three syndromes in Table 4. Concerning potential triggers, PHS could be triggered by dopaminergic drug reduction/discontinuation or STN-DBS power depletion [29, 31]. DHS could be triggered by dopaminergic drug increases [7, 10, 14] or dopaminergic drug changes (pramipexole IR to ER) [8]. SS is always triggered by serotonergic drug increases or combination of MAO-B and SSRI [33]. Notably, a high ambient temperature, infection, and trauma can induce both PHS and DHS. When patients are exposed to the above conditions, it is difficult to identify PHS and DHS only by triggers. Furthermore, it remains unclear why the same conditions triggered opposite movement disorders in DHS and PHS. Regarding disease duration, PHS and DHS commonly last from a few days to 1–2 weeks, while SS often lasts less than 24 h [33]. Furthermore, the neuromuscular signs of DHS commonly take the form of severe and generalized dyskinesia, while the neuromuscular signs of PHS and SS are often in the form of rigidity and akinesia [29, 35]. Moreover, SS is also accompanied by hyperreflexia, clonus, and myoclonus [35]. With regard to autonomic dysfunctions, PHS usually manifests as tachypnea, tachycardia, and hypertension or hypotension [27], while autonomic dysfunctions (except for hyperpyrexia) in DHS are rare. In addition to tachycardia, hypertension, and tachypnea, the autonomic dysfunctions of SS can also manifest as diarrhea, bowel sound hyperactivity, and mydriasis [35]. In terms of consciousness disturbance, PHS mostly presents as a reduced consciousness level, from drowsiness to coma [29]. However, DHS rarely develops into coma and often manifests as confusion and hallucinations. In addition, SS frequently occurs alongside anxiety and agitation in mildly to moderately affected patients and delirium or coma in severely affecting patients [35]. Removing the triggers is the most important treatment method for the three clinical syndromes. PHS warrants a gradual increase in dopaminergic drug dosage or restarting DBS [29]. In contrast, DHS warrants a gradual reduction in dopaminergic drug dosage or a reduction in DBS. Furthermore, SS warrants discontinuation of serotonergic drugs [35]. Besides acute treatment, prevention is also important to improve the prognosis. Prevention of these syndromes begins with an awareness of the differences and similarities between the three syndromes on behalf of clinicians. When these syndromes are identified, clinicians can carry out diagnosis and treatment quickly and correctly, avoiding any deterioration in a patient’s condition due to misdiagnosis. Moreover, the clinicians recognized the triggers of these syndromes. Clinicians should avoid all avoidable triggers (abruptly reduced anti-parkinsonian medications could trigger PHS, abruptly increased anti-parkinsonian medications could trigger DHS, and a combination of MAO-B and SSRI could trigger SS). In addition, when patients are exposed to unavoidable triggers (high ambient temperature and infection could trigger DHS and PHS), clinicians need to be aware of the possibility of these three syndromes. In order to quickly identify these three syndromes in clinical practice, we present a diagnosis flow chart (Fig. 4).

**Table 4:** Similarities and differences of PHS, DHS, and SS

	PHS	DHS	Serotonin syndrome
Potential triggers	Dopaminergic drug reduction or discontinuation^a^ STN-DBS dysfunction^a^ High ambient temperature Infection Trauma	Dopaminergic drug elevation^b^ High ambient temperature Infection Trauma	Serotonergic drug elevation^c^
Disease duration	Few days to 2 weeks	Few days to 2 weeks	Commonly within 24 h^c^
Neuromuscular signs	Rigidity^a^ Akinesia^a^ Tremor Dystonia Opisthotonos	Dyskinesia^b^	Rigidity Akinesia Tremor Clonus^c^ Hyperreflexia^c^ Myoclonus^c^
Autonomic dysfunctions	Tachycardia Tachypnea Hypertension Hypotension Sweating	Rare^b^	Tachycardia Tachypnea Hypertension Hypotension Sweating Diarrhea^c^ Bowel sounds activity^c^ Mydriasis^c^
Consciousness disturbance	Confusion Drowsiness Stupor or lethargy Coma	Confusion Hallucinations^b^ Stupor or lethargy Coma	Akathisia^c^ Anxiety^c^ Agitation^c^ Confusion Coma

^a^Clinical clues for diagnosing PHS. ^b^Clinical clues for diagnosing DHS. ^c^Clinical clues for diagnosing serotonin syndrome

## Conclusions

We summarized the triggers, clinical features, and treatments for all reported DHS cases and proposed guiding diagnostic criteria for DHS for the first time. In the future, it is clear that these diagnostic criteria still need to be further verified and revised by subsequently reporting DHS cases. In addition, we elucidated the similarities and differences between PHS, DHS, and SS, establishing a flow chart to guide diagnosis to quickly identify these three syndromes in clinical practice.

## Supplementary Information

Below is the link to the electronic supplementary material.

Supplementary file1 (DOC 64 KB)

## Abbreviations

PD: Parkinson’s disease
ER: Emergency room
PHS: Parkinson hyperpyrexia syndrome
DHS: Dyskinesia-hyperpyrexia syndrome
LEDD: Levodopa equivalent daily dose
NMS: Neuroleptic malignant syndrome
CK: Creatine kinase
STN-DBS: Subthalamic nucleus deep brain stimulation
SSRI: Selective serotonin reuptake inhibitors
MAO-B: Monoamine oxidase B

## References

[1] Ghosh R, Liddle BJ (2011). Emergency presentations of Parkinson's disease: early recognition and treatment are crucial for optimum outcome. Postgrad Med J.

[2] Gordon PH, Frucht SJ (2001). Neuroleptic malignant syndrome in advanced Parkinson's disease. Mov Disord.

[3] Richard IH, Kurlan R, Tanner C, Factor S, Hubble J, Suchowersky O, Waters C (1997). Serotonin syndrome and the combined use of deprenyl and an antidepressant in Parkinson's disease. Parkinson Study Group Neurology.

[4] Gil-Navarro S, Grandas F (2010). Dyskinesia-hyperpyrexia syndrome: another Parkinson's disease emergency. Mov Disord.

[5] Murad MH, Sultan S, Haffar S, Bazerbachi F (2018). Methodological quality and synthesis of case series and case reports. BMJ Evid Based Med.

[6] Haffar S, Bazerbachi F, Prokop L, Watt KD, Murad MH, Chari ST (2017). Frequency and prognosis of acute pancreatitis associated with fulminant or non-fulminant acute hepatitis A: A systematic review. Pancreatology.

[7] Lyoo CH, Lee MS (2011). Rhabdomyolysis induced by severe levodopa induced dyskinesia in a patient with Parkinson's disease. J Neurol.

[8] Taguchi S, Niwa J, Ibi T, Doyu M (2015). Dyskinesia-hyperpyrexia syndrome in a patient with Parkinson's disease: a case report. Rinsho Shinkeigaku.

[9] Herreros-Rodriguez J, Sánchez-Ferro Á (2016). Summertime Dyskinesia-Hyperpyrexia Syndrome. Clin Neuropharmacol.

[10] Acebrón Sánchez-Herrera F, García-Barragán N, Estévez-Fraga C, Martínez-Castrillo JC, López-Sendón Moreno JL (2017). Dyskinesia-hyperpyrexia syndrome under continuous dopaminergic stimulation. Parkinsonism Relat D.

[11] Baek MS, Lee HW, Lyoo CH (2017). A Patient with Recurrent Dyskinesia and Hyperpyrexia Syndrome. J Mov Disord.

[12] Sarchioto M, Ricchi V, Melis M, Deriu M, Arca R, Melis M, Morgante F, Cossu G (2018). Dyskinesia-Hyperpyrexia Syndrome in Parkinson's Disease: A Heat Shock-Related Emergency?. Mov Disord Clin Pract.

[13] Novelli A, Di Vico IA, Terenzi F, Sorbi S, Ramat S (2019). Dyskinesia-Hyperpyrexia Syndrome in Parkinson's disease with Deep Brain Stimulation and high-dose levodopa/carbidopa and entacapone. Parkinsonism Relat D.

[14] J Zu, HK Raza, T Chansysouphanthong, C Xu, W Zhang, G Cui (2021) Dyskinesia and hyperpyrexia syndrome: A case report and review of the literature. Rev Neurol (Paris) 18; S0035–3787 (20) 30739–6. 10.1016/j.neurol.2020.10.002. 10.1016/j.neurol.2020.10.002 33478739

[15] Ahlskog JE, Muenter MD (2001). Frequency of levodopa-related dyskinesias and motor fluctuations as estimated from the cumulative literature. Mov Disord.

[16] Prange S, Danaila T, Laurencin C, Caire C, Metereau E, Merle H, Broussolle E, Maucort-Boulch D, Thobois S (2019). Age and time course of long-term motor and nonmotor complications in Parkinson disease. Neurology.

[17] Blanchet PJ, Fang J, Hyland K, Arnold LA, Mouradian MM, Chase TN (1999). Short-term effects of high-dose 17beta-estradiol in postmenopausal PD patients: a crossover study. Neurology.

[18] Zappia M, Crescibene L, Arabia G, Nicoletti G, Bagala A, Bastone L, Caracciolo M, Bonavita S, Di Costanzo A, Scornaienchi M, Gambardella A, Quattrone A (2002). Body weight influences pharmacokinetics of levodopa in Parkinson's disease. Clin Neuropharmacol.

[19] Yamashita S, Uchida Y, Kojima S, Sakaguchi H, Kimura E, Maeda Y, Uchino M (2012). Heatstroke in patients with Parkinson's disease. Neurol Sci.

[20] Lin MT, Ho MT, Young MS (1992). Stimulation of the nigrostriatal dopamine system inhibits both heat production and heat loss mechanisms in rats. Naunyn Schmiedebergs Arch Pharmacol.

[21] Lin MT, Chandra A, Tsay BL, Chern YF (1982). Hypothalamic and striatal dopamine receptor activation inhibits heat production in the rat. Am J Physiol.

[22] de la Fuente-Fernández R (2007). Presynaptic mechanisms of motor complications in Parkinson disease. Arch Neurol.

[23] Linazasoro G (2007). Pathophysiology of motor complications in Parkinson disease: postsynaptic mechanisms are crucial. Arch Neurol.

[24] Canini F, Bourdon L (1998). Dopamine involvement in thermoregulatory responses to heat in rats. Neurosci Lett.

[25] Guatteo E, Chung KKH, Bowala TK, Bernardi G, Mercuri NB, Lipski J (2005). Temperature sensitivity of dopaminergic neurons of the substantia nigra pars compacta: involvement of transient receptor potential channels. J Neurophysiol.

[26] Tsai H-Y, Chen KC, Yang YK, Chen PS, Yeh TL, Chiu NT, Hui Lee I (2011). Sunshine-exposure variation of human striatal dopamine D2/D3 receptor availability in healthy volunteers. Prog Neuropsychopharmacol Biol Psychiatry.

[27] Petrulli JR, Kalish B, Nabulsi NB, Huang Y, Hannestad J, Morris ED (2017). Systemic inflammation enhances stimulant-induced striatal dopamine elevation. Transl Psychiatry.

[28] Toru M, Matsuda O, Makiguchi K, Sugano K (1981). Neuroleptic malignant syndrome-like state following a withdrawal of antiparkinsonian drugs. J Nerv Ment Dis.

[29] Harada T, Mitsuoka K, Kumagai R, Murata Y, Kaseda Y, Kamei H, Ishizaki F, Nakamura S (2003). Clinical features of malignant syndrome in Parkinson's disease and related neurological disorders. Parkinsonism Relat Disord.

[30] Gaig C, Marti MJ, Tolosa E, Gomez-Choco MJ, Amaro S (2005). Parkinsonism-hyperpyrexia syndrome not related to antiparkinsonian treatment withdrawal during the 2003 summer heat wave. J Neurol.

[31] Azar J, Elinav H, Safadi R, Soliman M (2019). Malignant deep brain stimulator withdrawal syndrome. BMJ Case Rep.

[32] Takubo H, Harada T, Hashimoto T, Inaba Y, Kanazawa I, Kuno S, Mizuno Y, Mizuta E, Murata M, Nagatsu T, Nakamura S, Yanagisawa N, Narabayashi H (2003). A collaborative study on the malignant syndrome in Parkinson's disease and related disorders. Parkinsonism Relat Disord.

[33] Boyer EW, Shannon M (2005). The serotonin syndrome. N Engl J Med.

[34] Isbister GK, Bowe SJ, Dawson A, Whyte IM (2004). Relative toxicity of selective serotonin reuptake inhibitors (SSRIs) in overdose. J Toxicol Clin Toxicol.

[35] Dunkley EJ, Isbister GK, Sibbritt D, Dawson AH, Whyte IM (2003). The Hunter Serotonin Toxicity Criteria: simple and accurate diagnostic decision rules for serotonin toxicity. QJM.

